# Field-based tests for determining critical speed among runners and its practical application: a systematic review

**DOI:** 10.3389/fspor.2025.1520914

**Published:** 2025-03-11

**Authors:** Lucie Lipková, Ivan Struhár, Jakub Krajňák, Dominik Puda, Michal Kumstát

**Affiliations:** ^1^ Department of Sport Performance and Exercise Testing, Faculty of Sports Studies, Masaryk University, Brno, Czechia; ^2^ Department of Physical Activities and Health Sciences, Faculty of Sports Studies, Masaryk University, Brno, Czechia

**Keywords:** critical power, endurance, testing methods, performance assessment, testing protocols

## Abstract

**Introduction:**

This review focuses exclusively on field-based critical speed (CS) tests for runners, aiming to evaluate key testing conditions to optimize field-based assessments and their practical applications.

**Methods:**

A systematic search was conducted in PubMed, Scopus, SPORTDiscus, and Web of Science databases in July 2024 using terms like “critical power,” “critical speed,” “testing,” and “field condition” along with related keywords. Following PRISMA 2020 guidelines, studies were systematically identified, screened, assessed for eligibility, and evaluated for the validity, reliability, and applicability of field-based methods for determining CS in runners.

**Results:**

From an initial pool of 450 studies, 19 met the inclusion criteria. The time trial (TT) test and the 3-minute all-out test (3MT) emerged as the most frequently used field-based methods, demonstrating high reliability when conducted under specific conditions.

**Conclusion:**

This review demonstrates that while field-based CS testing is a practical alternative to lab-based assessments, obtaining reliable results relies on following recommended testing settings, particularly for TT tests. By outlining the practical applications and conditions necessary for accurate CS assessment, this review supports athletes and coaches in applying CS testing effectively to enhance training strategies and performance.

## 1 Introduction

The critical power (CP) is a key parameter in understanding aerobic performance, defined as the asymptote of the power-duration relationship. It represents the highest power output (PO) an athlete can sustain without accumulating excessive fatigue (1, 2). In running, this model is mirrored by critical speed (CS), which reflects the maximum sustainable speed for prolonged efforts (3–6). Traditionally, CP has been considered a threshold distinguishing exercise intensity where physiological equilibrium can be maintained from those where it cannot, leading to fatigue manifestation (7–9). However, recent research (10, 11) highlights that CP is not a fixed threshold but rather a dynamic marker of fatigue, indicating the transition from sustainable to unsustainable exercise intensities.

Beyond CP, physiological homeostasis cannot be maintained, resulting in rapid exhaustion (10, 12, 13). This transition creates a “grey zone,” where metabolic responses such as lactate accumulation and oxygen consumption become increasingly complex as athletes approach and exceed CP (13). This nuanced understanding positions CP as more than a simple steady-state marker; instead, it indicates the point where metabolic instability begins to develop, signalling a loss of physiological control over exercise intensity (11). Above CP, the finite capacity for work is quantified by W′, representing the total amount of work an athlete can perform above CP before exhaustion occurs (14). In running, this is reflected by parameter D′, indicating the distance an athlete can cover above CS before the effects of fatigue culminate in exhaustion.

Once W′, or its running-specific counterpart D′, is depleted, athletes must reduce PO or speed to continue exercise (14, 15). Initially, W′ and D′ were considered an anaerobic source, such as intramuscular adenosine triphosphate (ATP), creatine phosphate (PCr), stored oxygen, and glycogen. More recent findings, though, suggest that W′/D′ is closely linked to the accumulation of fatigue-inducing metabolites, including lactate, inorganic phosphate (Pi), hydrogen ions (H+), and potassium (K+) (1, 7, 13). Managing these metabolites is essential for high-intensity, prolonged efforts, where pacing and energy reserve management are critical, particularly in middle- and long-distance running events (16).

While CP and CS are primarily used to optimize endurance performance, their application extends across a range of sports and activities, including interval training, fatigue monitoring, and performance evaluation. They are also applied to optimize recovery in team sports like rugby and hockey or to assess military fitness. This broader applicability underscores the flexibility of CP and CS, providing valuable insights not only for endurance athletes but across various disciplines. By refining training strategies and deepening the understanding of fatigue mechanisms, CP and CS contribute to optimizing performance, improving endurance management, and enhancing recovery strategies across multiple sports (17).

Determining CP and CS in both laboratory and field settings presents challenges. Reliable testing methods must balance validity and practicality. The time-to-exhaustion (TTE) test remains the benchmark for determining CP/CS and W′/D′, though it requires multiple tests at different constant POs, which can be time-consuming (18, 19). Alternative approaches, such as time trial (TT) tests over fixed distances or durations (9, 14, 16, 20) and the 3-minute all-out test (3MT), which is designed to fully deplete W′ in the first 150 s while maintaining a PO equal to CP in the final 30 s (2, 9, 15), offer more practical solutions. Despite their efficiency, these methods have limitations in reliability and accuracy.

Field-based assessments of CS, introduced in the 1990s, provided practical alternatives to laboratory tests. However, their accuracy was initially limited due to environmental variability, inconsistent methodologies, and a lack of standardized protocols (21). Over time, advancements such as wearable technology, improved mathematical models, and better data collection techniques have enhanced the precision of field assessments. Despite these improvements, selecting protocols that balance validity, reliability, and ease of application remains a significant challenge, particularly for use in competitive and training settings (22, 23).

Although the models of CP and CS have been extensively studied in cycling (24, 25) and swimming (26, 27), their application in running has gained increasing attention due to its potential for performance optimization. However, applying them in running introduces unique challenges due to environmental factors and protocol variability. This review aims to address these challenges by focusing exclusively on field-based CS tests for runners, assessing their practical applications, key testing conditions, and evaluating their strengths and weaknesses. Additionally, it explores practical applications of the CS, providing actionable insights for athletes and coaches to implement these tests in training and performance optimization.

## 2 Methods

### 2.1 Search strategy

The systematic review followed the 2020 updated Preferred Reporting Items for Systematic Reviews and Meta-Analyses (PRISMA) guidelines (28). The search strategy was performed (July 2024) in PubMed, Scopus, SPORTDiscus, and Web of Science. The selected keywords and Boolean operators for the search were: (“critical power” OR “critical speed” OR “critical velocity”) AND (“test” OR “tests” OR “testing” OR “method” OR “methods”) AND (“running” OR “runners” OR “runner” OR “athletes” OR “athlete”) AND (“field” OR “track” OR “field condition”).

### 2.2 Selection of studies and results extraction

All records retrieved from the database search were imported into the Rayyan systematic review software (29) for screening. Two reviewers (DP and JK) independently conducted the screening process, and any disagreements were resolved by consultation with a third reviewer (LL). Exclusion criteria included non-English language publications, reviews, meeting abstracts, letters, corrections, editorials, and non-human studies. Titles and abstracts were initially screened to exclude studies lacking the predefined keywords. The inclusion of articles was restricted to those published between 2010 and 2024. Studies were further assessed based on the PECO criteria (Table 1), with eligibility determined during the full-text review. Studies focused solely on novice or untrained individuals were excluded at this stage to ensure applicability to athletic populations. Additionally, studies with fewer than six participants were excluded to minimize deviations due to small sample sizes (30). Data extraction was conducted by one reviewer (LL) and cross-verified by a second reviewer (MK) where necessary.

**Table 1:** PECO criteria.

PECO criteria	Inclusion criteria
P = participants	Female or male athletes, recreational to elite runners, age more than >18
E = exposure	Field-based protocols for determining CS: including single-visit methods (e.g., 3MT), multi-visit methods (e.g., TTs or TTE tests), CS estimation derived from training or competition data
C = comparison	Comparison of different field-testing methods (e.g., single-visit vs. multi-visits), comparison of filed-based method with laboratory tests (e.g., treadmill protocols), or evaluation of predictive accuracy for performance outcomes (e.g., race times, endurance capabilities)
O = outcome	Accurate determination of CS and related parameters (e.g., W′/D′). Validation of field test settings (e.g., time/distance/power/speed metrics). Test reliability (e.g., test-retest coefficients). Practical utility for training or performance prediction.

### 2.3 Assessment of study quality

The assessment of study quality utilised the Downs and Black scale (31). The original checklist includes 27 questions assessing the quality of reporting, internal and external validity, and statistical power. For this review, the checklist was adapted by excluding 12 questions that were not relevant to the selected studies, such as those specific to intervention designs. As a result, 16 questions were used to evaluate the studies, focusing on reporting clarity, study design validity, and appropriate statistical analysis (Supplementary Table S1). Two reviewers independently conducted the quality assessment, with disagreements resolved by a third reviewer. Each item received a binary score, with 1 representing “yes” and 0 indicating “no” or “unable to determine.” The total points were converted to percentages and adjusted according to the number of selected questions. In this review, the Downs and Black (31) total score was adjusted to a maximum of 15 points, as one question was considered self-evident and not included in the scoring. Studies scoring less than 45.4% (6 points or fewer) were categorized as having “poor” methodological quality. Scores between 45.4% and 63.6% (7–8 points) indicated “fair” quality, while scores above 63.6% (9 points or more) reflected “good” methodological quality.

## 3 Results

**Figure 1:**
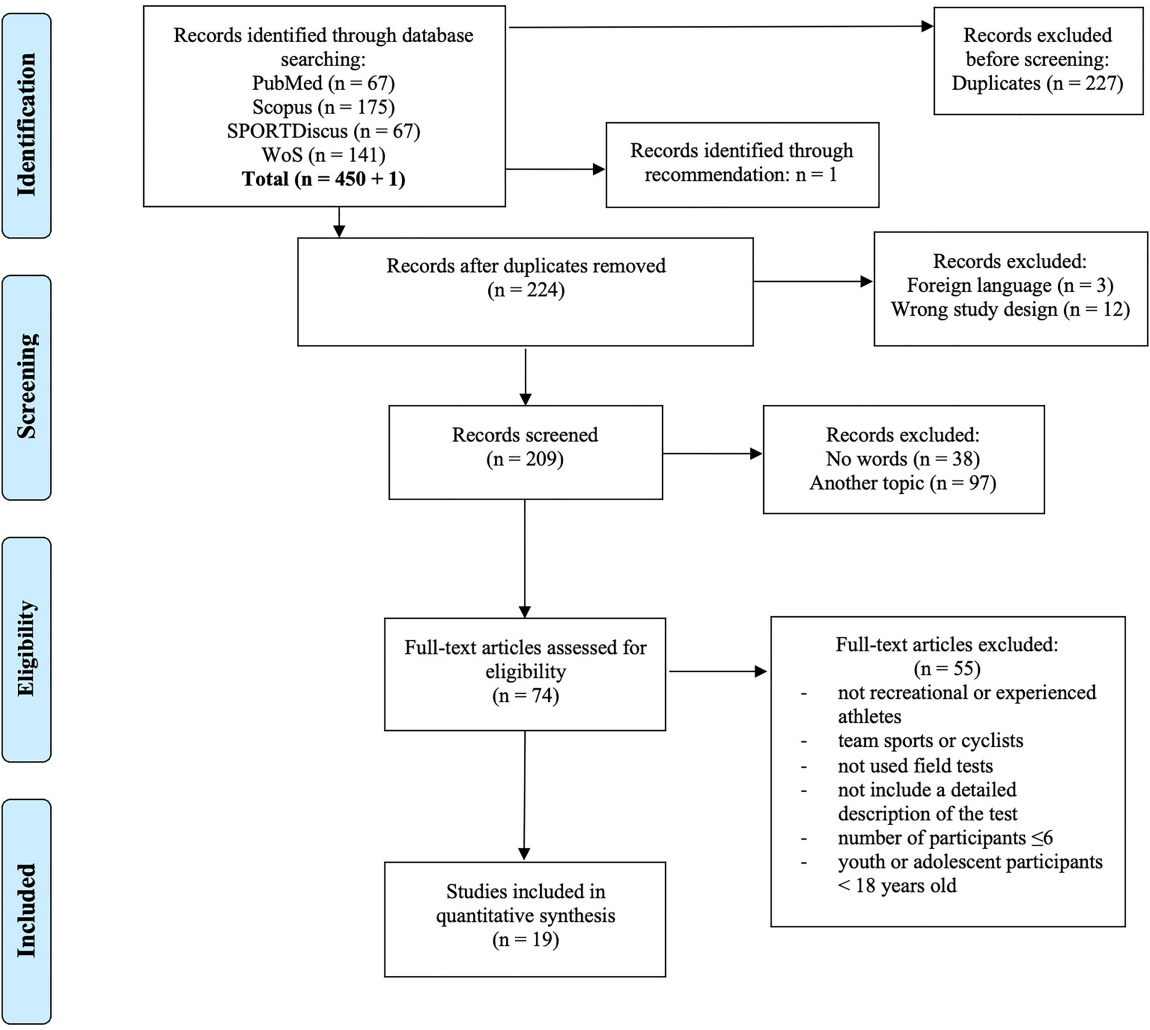
PRISMA flow diagram of the search strategy.

A total of 450 studies were initially identified, with 227 duplicates removed prior to the screening process (Figure 1). One additional studies was included based on recommendations. Furthermore, 15 studies were excluded due to factors such as non-English language, wrong study design (e.g., reviews, meeting abstracts, letters, editorials), or non-human subjects. Subsequently, 135 articles were excluded following the screening of titles, abstracts, keywords, and topics. A total of 74 full-text articles underwent further assessment, resulting in the selection of 19 studies that met all predefined PECO criteria (Table 1). These studies provided detailed descriptions of field-testing protocols and evaluated the validity, reliability, or predictability of CS and related measures.

### 3.1 Characteristics of the participants

Table 2 summarises the main characteristics of participants from the included studies. A total of 285 participants contributed to this research (207 males, 66 females, mean ± SD: age = 30.6 ± 6.3 years). Smyth and Muniz-Pumares (41) analyzed a dataset of 31,190 runners from Strava®; however, due to the large sample size and its focus on aggregated training data, this study was not included in these statistics. Ten studies included only male participants, while seven had a mixed sample, and only one study focused solely on female participants. The limited representation of female participants may influence the generalizability of the results, which should be considered in further analysis. The participants’ training experience varied widely, ranging from as little as 6 months to over 18 years. Training volumes also differed, from 4 h per week to 110 km per week, encompassing a mix of recreational, regional, and national-level runners.

**Table 2:** Participant characteristics across included studies.

Author	*n*	Sex (F, M)	Age (mean ± SD)	Subjects	VO_2max_ (ml·kg^–1^ min^–1^, mean ± SD)	Training/experience/level (years)
Aguiar et al. (14)	7	M	26 ± 5	College students	56.6 ± 4.1	2x/wk running
Broxterman et al. (15)	7	4 M, 3 F	25.3 ± 3.4	Healthy subjects	49.6 ± 5.7	ranged from not active to highly trained (<150 min marathon)
Corrêa et al. (20)	34	20 M, 14 F	42.4 ± 11.0	Runners	NO	18 years running; participation in at least one 10-km race
Figueiredo et al. (32)	25	M	28.6 ± 4.7	Runners	NO	recreational, regional and local
Galbraith et al. (33)	10	M	39 ± 7	Middle-distance runners	60.7 ± 2.8	competitive club standard; 2 years of competing
Galbraith et al. (34)	13	M	33 ± 14	Middle/long-distance runners	No	min 3 years competing
Galbraith et al. (35)	14	M	28 ± 8	Runners	69.8 ± 6.3	11 years of running training, national level
Kordi et al. (36)	14	M	28 ± 8	Distance runners	69.8 ± 6.3	11 ± 2 years running; local athletic clubs
Olaya-Cuartero et al. (37)	9	M	38.1 ± 5.4	Runners	NO	>150 min/w, half-marathon time 1:25:36 ± 00:11:20
Pettitt et al. (38)	14	F	19 ± 1	Distance runners	55 ± 4	NO
Ribeiro et al. (39)	34	20 M, 14 F	42.4 ± 11.0	Amateur runners	49.5 ± 8.1	>6 months uninterrupted training; participation in at least one 10-km race
Ruiz-Alias et al. (40)	15	8 M, 7 F	23 ± 5	Trained athletes	NO	110 ± 15 km/week, 5 km best 15:29 ± 00:53
Ruiz-Alias et al. (23)	15	8 M, 7 F	23 ± 5	Trained athletes	NO	110 ± 15 km/week, 5 km best 15:29 ± 00:53
Smyth and Muniz-Pumares (41)	31.190	M, F	39 ± 8	Runners	NO	Frequency of activities per week 3.6, volume 41.3 (km per week)
Triska et al. (42)	10	M	31.0 ± 5.7	Endurance-trained triathletes	NO	8 h/wk running; 3 years
Triska et al. (16)	10	M	24.9 ± 2.1	Moderately trained	52.9 ± 3.1	4 h/wk running
Van Rassel et al. (22)	10	7 M, 3 F	29 ± 7	Runners	59.0 ± 4.2	Recreationally active or trained/developmental runners
Vassallo et al. (43)	9	M	24 ± 3	Healthy active	4.4 ± 0.5*	NO

F = Female, M = Male, SD = standard deviation, NO = value not mentioned, S_max_ = maximal speed, wk = week.

*values measured in liter per minute (L·min^–1^).

### 3.2 Characteristics of the field tests

The research included a variety of field-based running test procedures to evaluate CS/CP, reflecting the diversity in approaches across studies (Table 3). The TT were the most popular method, involving fixed distances (iso-distance) ranging from 400 to 3,600 m (14, 16, 20, 22, 23, 32–36, 39, 40, 42), with the most popular over three fixed distances: 1,200, 2,400 and 3,600 m (22, 33–36). Some studies employed fixed-duration trials (iso-duration) (23, 40, 42, 44). The 3MT (14, 15, 38, 43, 44) challenged participants to achieve maximum speed over a brief period, offering an alternative perspective on performance. Hunter et al. (44) explored the application of 3MT and TTs alongside habitual training (HAB) data, demonstrating the potential for estimating CS/CP from non-invasive and remote methods. Similarly, Smyth and Muniz-Pumares (41) analyzed large-scale activity datasets logged on Strava®, offering an innovative approach to derive CS and CP from everyday training records.

**Table 3:** Description of running test protocols in included studies.

Author	Num. of trials	Protocol (outcome)	Setting	Critical speed (m.s^−1^)/power (watts) (mean ± SD)	Compared with laboratory tests/with performance	Recovery time between sessions	Calculation (Math. model)	Condition
Aguiar et al. (14)	3	TT	800, 1,600, 2,400 m	3.69 ± 0.50	NO	at least 48 h	Linear distance-time	200 m synthetic outdoor track
				3.77 ± 0.47			Linear inverse-of-time	
	1	3MT	As fast as possible	Test 3.90 ± 0.41		—	CS over the last 30 s	
				Retest 3.89 ± 0.48				
Broxterman et al. (15)	1	3MT	As fast as possible	13.4 ± 2.8*	13.3 ± 2.8* (TTE—treadmill linear inverse-of-time)	—	CS over the last 20 s	running track
Corrêa et al. (20)	2	TT	400, 2,000 m (as fast as possible)	13.9 ± 2.2*	13.4 ± 2.1 (10 km race)	48 h	Linear distance-time	400 m running track
Figueiredo et al. (32)	3	TT	2,600, 1,800, 1,000 m	12.1 ± 1.4	12.0 ± 1.3 (5 km run)	30 min	Linear distance-time (combination of 2 or 3 TTs)	400 m running track (completion times 3–12 min)
Galbraith et al. (33)	3	TT	3,600, 2,400, 1,200 m	4.07 ± 0.28	4.05 ± 0.22 (TTE –treadmill linear distance-time)	30 min	Linear distance-time	400 m running track (12-, 7- and 3-min completion time)
				4.07 ± 0.26		60 min		
Galbraith et al. (34)	3	TT	3,600, 2,400, 1,200 m	4.41 ± 0.48	NO	30 min	Linear distance-time	400 m running track
Galbraith et al. (35)	3	TT	3,600, 2,400, 1,200 m	4.90 ± 0.32	compared with VO_2max_, lactate threshold and running economy	30 min	Linear distance-time	400 m athletic track (average temperature 13.8 °C, wind speed ≤2.0 m/s)
				4.99 ± 0.30				
Hunter et al. (44)	3	TT	3, 7 and 12 min	CS: 3.42 ± 0.53 m·s^−^¹; CP: 290 ± 44 W	NO	24 h	Hyperbolic model, linear distance/power-time, linear inverse-of-time	all test on the same route, with minimal changes in elevation and sharp corners (Stryd power meter)
	1	3MT	as fast as possible	CS: 3.76 ± 0.57 m·s^−^¹; CP: 305 ± 53 W, CS: 3.77 ± 0.60 m·s^−^¹; CP: 307 ± 52 W		—	CS over the last 30 s	
	Multiple	Habitual training data	—	CS: 3.44 ± 0.63 m·s^−^¹; CP: 281 ± 41 W		—	Hyperbolic model, linear distance/power-time, linear inverse-of-time	
Kordi et al. (36)	2	TT	3,600 and 1,200 m	4.94 ± 0.32	NO	30 min	Linear distance-time	400 m running track
	3		3,600, 2,400 and 1,200 m					
Olaya-Cuartero et al. (37)	1	TT (9/3-min Stryd test	9 min maximum effort, 3 min	4.45 ± 0.67	4.32 ± 0.57 (half-marathon)	30 min	Stryd CP calculator	400-m running track (Stryd running power meter)
Pettitt et al. (38)	1	3MT	As fast as possible	4.46 ± 0.41	4.55 ± 0.24 (50%Δ treadmill GXT)	—	CS over the last 30 s	running track (using GPS)
Ribeiro et al. (39)	2	TT	400, 2,000 m	13.9 ± 2.2	13.4 ± 2.1 (10 km race)	48 h	Linear distance-time	running track (400 m)
Ruiz-Alias et al. (23)	2	TT	3, 9 min	338 ± 55	230 ± 43 (TT—3,9 min treadmill)	30 min	Work-time, inverse-of-time	400 m running track (Stryd power meter)
				319 ± 52 (no wind)				
Ruiz-Alias et al. (40)	5	TT	3, 4, 5, 10, 20 min	263 ± 58	NO	72 h	Linear work-time	400 m running track (Stryd power meter)
				265 ± 59			Linear inverse-of-time	
Smyth and Muniz-Pumares (41)	Multiple	Raw training data	Fastest times for 400, 800, 1,000, 1,500, 3,000, 5,000 m within 16 weeks pre-marathon	3.74 ± 0.08 m·s^−^¹	Marathon performance (*R*^2^ = 0.67)	—	Linear distance-time	Strava® online platform
Triska et al. (16)	3	TT	TTs equalled the corresponding time to TTE trials	3.77 ± 0.35	3.75 ± 0.36 (TTE—treadmill)	48 h	Linear inverse-of-time	400 m running track
Triska et al. (42)	3	TT	12, 7 and 3 min	4.17 ± 0.37	NO	60 min	Linear inverse-of-time	400 m running track
			10, 5 and 2 min	4.29 ± 0.30				
Van Rassel et al. (22)	2	TT	1,200, 2,400 m	3.97 ± 0.42 (CS)	Speed at CP (800 m) = 3.95 ± 0.46	60 min	Linear inverse-of-time	400 m running track
				278 ± 29 (CP)	Power at CP (800 m) = 275 ± 29			
	3		1,200, 2,400 m + (1 of 3,600, 4,000, 4,400 m to cover ∼15 min)	3.89 ± 0.44 (CS)	Speed at CS (800 m) = 3.88 ± 0.44			
				270 ± 28 (CP)	Power at CS (800 m) = 271 ± 28			
Vassallo et al.	4	TTE	Running speeds attained at VO_2max_, 110% VO_2max_, Δ70% (difference between GET and VO_2max_), Δ85% (exhaustion within 2 and 15 min)	3.7 ± 0.2	NO	48 and 72 h	Linear distance-time	400 m running track (using audio tone to control speed)
				424 ± 20			Linear work-time	
	1	3MT	As fast as possible	Test (3.6 ± 0.4)		NO	CS over the last 30 s (speed-time)	400 m running track (power and speed derived from GPS)
				Retest (3.6 ± 0.4)				
				Test (443 ± 37)			CP over the last 30 s (power-time)	
				Retest (450 ± 36)				

* km.h^−1^ *comparing with linear test; 3MT, 3 min all-out test; CS, critical speed; GET, gas exchange threshold; TT, time trial; TTE, time to exhaustion test (constant-work rate); T10, 10-minute submaximal treadmill test; VO_2max_, maximum oxygen uptake; Δ, magnitude of the interval between gas exchange/ventilatory threshold and VO_2max_/maximal aerobic running speed.

In addition to these methods, Vassallo et al. (43) applied the gold-standard TTE test in a field setting and introduced an enhanced 3MT model that incorporates energetic calculations, offering a practical approach to estimate CP and CS in outdoor conditions with power derived from GPS and speed variations accounted for. Notably, Olaya-Cuartero et al. (37), Ruiz-Alias et al. (23), Hunter et al. (44) and Van Rassel et al. (22) utilized the Stryd power meter to determine CP, either through the 9,3-minute CP test (23, 37) or via the iso-distance model (22). Several mathematical models were employed for CS determination, including linear distance-time relationships (e.g., Linear-TD), inverse-of-time models (e.g., INV), work-time calculations (e.g., linear work-time), and hyperbolic models (e.g., 2-HYP). Some studies also compared field test results with laboratory-based protocols (15, 16, 23, 33, 35, 38), as well as with performance outcomes, enhancing the assessment of the validity and reliability of these methods.

To summarize, the field-based methods for determining CS/CP were grouped into three main categories: TTs (including traditional iso-distance and iso-duration trials, as well as innovative approaches like habitual activity tracking and raw race data analysis), TTE, and 3MT.

### 3.3 Assessment of study quality

The study quality was evaluated based on a maximum score of 15 points. Five studies achieved the score of 12 (80%), seven studies scored 11 (739%), four studies scored 10 (66%), and the remaining four studies scored 9 or 8 (60%–53%). No studies scored below 8, indicating generally fair to good methodological quality across all included studies (Supplementary Table S2).

## 4 Discussion

This systematic review provides insights into field-based assessments of CS for runners, with a focus on the key settings and practical application of various testing protocols. The findings highlight the use of TTs over varying distances and durations, as well as the 3MT, which are commonly employed in real-world scenarios. The effectiveness of these methods is influenced by factors such as the number of trials, their duration or distance, recovery time between trials, and trial order. Additionally, innovative approaches such as HAB tracking and raw race data analysis have expanded the possibilities for estimating CS without requiring dedicated testing sessions. By addressing these factors and exploring novel approaches, this review offers actionable insights for athletes and coaches to effectively integrate CS testing into training and performance optimization.

### 4.1 Traditional and TTs key setting

In endurance research, two primary testing methods exist: the traditional TTE, where participants run at a constant speed over several trial (3–5) until exhaustion and the TTs, where athletes cover a fixed distance or run for a set duration at their maximal intensity. While the TTE is effective in controlled environments, they are less practical for field applications due to the difficulty of maintaining consistent pacing. For instance, Vassallo et al. (43) conducted a TTE test using audio tone for pace synchronization, ending the test when the athlete could no longer meet the required speed. However, even with auditory guidance, verifying that participants truly exert maximal effort remains challenging in the absence of physiological markers like VO_2max_ attainment. Moreover, environmental factors, such as wind, temperature, or uneven terrain, can further complicate pacing and reliability, making TTE a less optimal method for field conditions. These limitations underscore why TTE tests are less favored for field assessments compared to other methods.

The choice of mathematical model has a key influence on CS and D′ estimates. Linear models (INV, EXP) typically provide higher CS values, while non-linear models (2-HYP, 3-HYP) are more reliable for estimating D′ but exhibit greater variability. These differences should be carefully considered when selecting the number and duration of trials, as inaccuracies can be a source of variability in performance predictions. While mathematical models are not the primary focus of this review, they are extensively discussed in other studies (45–48), providing deeper insights into their application and limitations.

#### 4.1.1 Trial duration and length

In contrast, TTs have become the preferred method for field testing, as they more accurately reflect real-world exercise conditions and offer greater flexibility in trial design. However, due to the considerable variability in testing settings, careful consideration must be given to selecting the most appropriate protocol for specific objectives and contexts. Trials typically fall within a 3–12 min time window (e.g., 3,600, 2,400, and 1,200 m), corresponding to exhaustion times of 3, 7, and 12 min (22, 33–36, 42, 44). This time range balances the engagement of both aerobic and anaerobic systems while maintaining sufficient intensity to approach VO_2max_. Time-based protocols are particularly advantageous as they provide more consistent physiological markers, reducing variability introduced by individual differences in speed or fitness levels.

Shorter trials have been shown to yield higher CS values compared to longer ones, emphasizing the impact of trial duration on the accuracy of CS estimation. Triska et al. (42) demonstrated that trials lasting 10, 5, and 2 min produced higher CS values than those lasting 12, 7, and 3 min. Similarly, Galbraith et al. (33), using the same longer protocol (12, 7, and 3 min), found that while CS measurements remained reliable, D′ exhibited a 13.3% variation, underscoring its sensitivity to trial duration. These findings highlight the importance of carefully selecting trial durations that balance the physiological demands of anaerobic and aerobic systems to ensure reliable results.

This variability reflects the metabolic demands associated with trial duration. Shorter trials, due to their high intensity, predominantly rely on anaerobic metabolism, often leading to incomplete depletion of D′ and inconsistent attainment of VO_2max_. In contrast, longer trials (>15–20 min) may fail to sustain the severe-intensity domain due to reduced intensity or motivational factors, leading to inconsistent attainment of VO_2max_ (17, 49). VO_2max_ is rarely achieved in trials shorter than 1–2 min or longer than 15–20 min, highlighting the importance of optimizing trial duration for accurate assessment (11, 50). Caen et al. (51) emphasized the necessity of adhering to strict methodological criteria for CP/CS determination in cycling, including trial durations between 2 and 15 min and ensuring VO_2max_ attainment. While these findings were developed in the context of cycling, the shared physiological principles underlying endurance performance suggest that similar practices could enhance reliability and minimize variability in field-based CS estimates for runners.

Beyond the complexities of selecting an optimal trial duration, achieving and verifying VO_2max_ during field testing remains a critical challenge. While field tests closely mirror laboratory results, the absence of physiological markers like VO_2max_ introduces uncertainty. Portable metabolic analyzers, such as the wearable Cortex device, offer a potential solution by enabling VO_2max_ validation during field trials. However, integrating such equipment increases complexity and costs, potentially limiting accessibility, especially for recreational athletes or resource-constrained settings. Nevertheless, adopting such technologies could enhance the reliability of CS estimates across diverse environments, ensuring their applicability for both research and practical training purposes.

#### 4.1.2 Number of trials and predictive accuracy

The number of trials required for field-based CS testing is a critical consideration in test design. While three trials are commonly employed to ensure accuracy, this approach can significantly increase the time burden of testing. Consequently, some studies have investigated whether two trials could provide comparable reliability. Figueiredo et al. (32) found no significant differences between CS values derived from a three-TT protocol (2,600, 1,800, and 1,000 m) and a two-TT protocol (2,600 and 1,000 m). Similarly, Gifford and Collins (52) confirmed that CS calculated from 1,500 and 3,000 m trials strongly correlated with true CS. Kordi et al. (36) further demonstrated that two-point protocols (3,600 and 1,200 m) were as effective as more complex three-point versions for predicting 5 km performance. Notably, the three-point model for 5 km performance prediction showed a higher correlation with CS than peak running velocity in recreational runners (32).

The use of two trials, however, remains a subject of debate. Pethick et al. (12) cautioned that two trials might not fully account for standard error, potentially introducing bias in D′ estimation when greater precision is required. Ruiz-Alias et al. (40) and Van Rassel et al. (22) further demonstrated that two trials using the Stryd power meter provided comparable CS and CP values to those from three trials, suggesting that a third trial may not be necessary. It is important to note, however, that the accuracy of the Stryd power meter can depend on where the device is placed—most commonly on the laces of the right foot. This placement may introduce a biological error linked to the device's location (i.e., left or right limb), particularly relevant for track athletes who often display asymmetries in strength and muscle stiffness between lower limbs (23). These findings highlight the need to carefully balance the trade-offs between achieving accuracy and maintaining practicality in field-testing protocols, while also accounting for potential sources of error, such as equipment placement and individual variability.

Field-based tests have also been shown to offer superior predictive accuracy for outdoor race performance compared to laboratory-based tests. Ruiz-Alias et al. (23) reported that track-based CP and W′ values were significantly higher than those from treadmill tests, even when adjusting for wind resistance. This finding underscores the physiological differences between testing environments and the potential advantages of field-based protocols in reflecting outdoor performance. As an example, Nimmerichter et al. (6) found that treadmill-based CS estimates tended to underestimate 5 km race performance by 5%–9%, whereas field-based tests over fixed distances (400 and 2,000 m), such as those validated by Corrêa et al. (20) and Ribeiro et al. (39), proved more reliable for predicting 10 km race velocities. These studies collectively suggest that field-based tests not only yield higher performance metrics but also offer greater predictive accuracy for real-world race outcomes.

However, some limitations persist. For instance, Corrêa et al. (20) identified biases in performance prediction, with men tending to overestimate and women to underestimate their capabilities, highlighting the limited representation of female participants in these studies. This gap restricts the generalizability of findings across genders. Furthermore, the use of shorter distances, such as 400 m, deviates from the more commonly employed protocols and may influence the results by skewing estimates towards higher speeds.

Balancing the number and duration of trials is critical for designing effective field tests. Two-trial protocols, while practical for time-constrained training settings, remain a topic of debate, as some researchers argue they may compromise precision in estimating CS and D′. In contrast, three-trial protocols are increasingly preferred due to their ability to deliver greater accuracy and reliability, making them the more robust choice in most scenarios.

#### 4.1.3 Order, time gap, and recovery time in protocol design

In addition to trial duration and length, the order, gap and recovery between trials significantly affect outcomes. Ruiz-Alias et al. (40) recommended a minimum gap of 7 min between the shorter and longer trials, as shorter gaps (e.g., 3 and 4 min) did not meet validity criteria. In contrast, combinations such as 3–10 min or 5–20 min produced valid results. Additionally, trial order was found to influence PO, with longer trials conducted first yielding higher outputs (40). Most studies favor arranging trials from longest to shortest or randomizing the order to minimize potential bias.

Equally important is the recovery time between trials, which also influences the protocol design. While longer recovery periods (e.g., 3 h and 30 min) (23, 32–36, 42) are typically recommended for ensuring high agreement and low prediction error in CP assessments, recent studies suggest that shorter recovery periods, such as 30 min, may be sufficient in running protocols (23, 32–34, 36). Triska et al. (53) found that a 30-min recovery produced similar POs compared to tests conducted on different days. Moreover, CS values from 30- or 60 min recovery field tests correlated well with treadmill-based tests, though discrepancies were noted in D′ values between treadmill and field protocols (33).

These findings highlight the importance of tailoring trial recovery times to the context of field testing. Shorter recovery periods offer a practical solution for time-constrained settings, particularly in training environments, without significantly compromising performance reliability. However, careful consideration of trial order and randomized sequences remains crucial for minimizing potential biases and ensuring valid results.

#### 4.1.4 Innovative approaches and emerging technologies

Innovative methodologies have expanded the scope of field-based CS and CP estimation. Hunter et al. (44) utilized three TTs (3, 7, and 12 min) in uncontrolled conditions, supplemented by power meter technology (Stryd Inc.), and incorporated a HAB approach, in which participants tracked their regular training over six weeks. Retrospective analyses revealed no significant differences in CS and CP estimates among TT, 3MT, and HAB methods, even when accounting for environmental variability. Despite these promising results, questions remain about the optimal duration of HAB data collection and the extent to which environmental factors, such as weather and terrain, influence CS accuracy over prolonged periods.

Similarly, Smyth and Muniz-Pumares (41) utilized raw race data from distances of 400, 800, 1,000, 1,500, 3,000, and 5,000 m to estimate CS. The best-performing model, incorporating 400, 800, and 5,000 m distances, achieved a low prediction error (∼7.67%) for marathon performance, demonstrating its utility in race planning and analysis. Despite these promising outcomes, the reliance on race data, like the HAB approach, presents limitations. The inability to verify maximal effort introduces variability, which may compromise the robustness of CS estimates in certain scenarios. Additionally, environmental conditions, such as weather and terrain, along with motivational factors, can influence the reliability of these methods.

Nevertheless, HAB and raw data approaches offer a seamless integration into training routines without requiring additional testing sessions. They are particularly advantageous for monitoring performance trends over time in competitive athletes. However, these methods require careful consideration of variables such as maximal effort, motivation, and environmental consistency, which may vary across observation periods.

### 4.2 3-minute all-out test

The 3MT is a valuable alternative to traditional TTE and TTs, offering a more time-efficient way to estimate CP and CS without requiring multiple trials. Its design is grounded in the principle of fully depleting the anaerobic reserve (D′) within the first 150 s of an all-out effort, after which the power output or speed stabilizes. This stabilization phase reflects the athlete's CS, representing the maximum sustainable speed without further depletion of D′ (14, 15). This stabilization represents the transition from anaerobic to aerobic energy pathways.

The simplicity and practicality of 3MT has been enhanced by tools such as GPS devices, accelerometers, power meters and stopwatches, enabling its use in field settings (14, 15, 43, 44, 54). Vanhatalo et al. (55) demonstrated that these technologies facilitate the application of 3MT in real-world conditions, making it a valuable tool for athletes and coaches. However, transitioning from controlled laboratory settings to outdoor environments introduces challenges, including surface variability, wind, temperature, and athlete motivation. These factors, combined with the potential for pacing behaviors, underscore the importance of robust protocol design. Hunter et al. (44) found that despite instructions, pacing behaviors were observed, highlighting the need for careful participant preparation and protocol design.

Studies have consistently validated the 3MT for estimating CS. Aguiar et al. (14) reported high reliability for CS determination, with test-retest coefficients above 0.90, though the test underestimated D′ by approximately 16%. Similarly, Broxterman et al (15). found a strong correlation (*r* = 0.92) between field-based 3MT and treadmill-based TTE tests, further confirming the validity of 3MT in estimating CS. Although W′ was again underestimated. Pettitt et al. (38) demonstrated that GPS-enabled 3MT provides reliable predictions for races ranging from 1,600 to 5,000 m, although errors increased for shorter events like the 800 m.

Innovations in 3MT methodology have aimed to address some of its limitations. Vassallo et al. (43) introduced a novel power-based model that tracked real-time variations in power output during over-ground running. By focusing on power rather than speed, this approach accounted for pacing fluctuations, acceleration, and deceleration, providing a more comprehensive assessment of mechanical demands. Although this model tended to overestimate CP and D′ compared to traditional protocols (by approximately 25 W for CP and 7 kJ for D′), it highlighted the potential for integrating power-based measurements into 3MT applications, particularly for sports requiring frequent speed changes. Another innovative approach was demonstrated by Hunter et al. (44), who showed that 3MT could be conducted unsupervised with reliable outcomes when participants followed clear instructions. However, this adaptation raises questions about whether unsupervised results are comparable to those obtained under supervised conditions. Future research should focus on establishing the reliability and validity of unsupervised 3MT across diverse field environments, particularly for recreational athletes.

While the 3MT offers significant advantages in terms of practicality and efficiency, it appears better suited for experienced runners accustomed to performing at maximal intensity. Novice runners may struggle to deliver consistent all-out efforts, potentially affecting test accuracy. Additionally, field tests lack the ability to verify maximal efforts through physiological markers like VO_2max_ attainment, which adds complexity to standardizing results. The consistent underestimation of D′ presents challenges for prescribing high-intensity interval training, emphasizing the need for careful protocol design. Environmental factors, such as surface variability and weather conditions, further highlight the importance of controlled settings to ensure reliable outcomes.

In conclusion, the 3MT integrates anaerobic and aerobic parameters in a single trial, making it a valuable tool for estimating CS in both laboratory and field settings. Continued advancements, such as the integration of power-based monitoring and innovative technologies, hold promise for enhancing the test's accuracy and applicability. However, its dependence on maximal effort and sensitivity to pacing behaviors highlight the importance of robust protocols, participant preparation, and environmental consistency to optimize test reliability.

### 4.3 Practical applications of the critical speed concept

The CS is a valuable tool for assessing endurance performance and planning effective training strategy for runners. However, applying CS in real-world settings presents unique challenges, particularly due to variability in physiological responses around the CS threshold. This variability creates a “grey zone,” as described by Jones et al. (11), where uncertainty exists near the estimated CS. For example, a CS value of 5 m/s with a 5% error margin could place an athlete either in the heavy-intensity domain (below CS at 4.75 m/s) or the severe-intensity domain (above CS at 5.25 m/s), complicating training prescriptions and pacing strategies. Caen et al. (51) also emphasized that such variability is influenced not only by biological factors but also by methodological aspects, including differences in trial durations and testing protocols. Addressing these factors is essential to refine CS testing protocols, ensuring greater accuracy and applicability in both research and practical training settings.

Studies have validated the robustness of CS for training purposes. Figueiredo et al. (56) demonstrated that CS and peak running velocity are equally effective for prescribing endurance training in recreational runners when determined under controlled track settings. At the same time, gender-specific differences in pacing strategies highlight the practical relevance of CS. Female marathon runners, for example, tend to perform closer to their CS than males, particularly in time brackets of 170–360 min. This even pacing approach contrasts with the variability observed in male runners, who often experience significant slowdowns in the latter stages of a race, especially those with slower performances. For less experienced runners, individualized pacing strategies based on CS are essential to mitigate fatigue and sustain performance (41).

In competitive scenarios, the utility of CS extends to performance prediction and race strategy planning. Elite marathoners typically compete at approximately 95% of their estimated CS (57), while half-marathon runners have been shown to race at around 97.3% of their CP, with no significant differences between CP determined by the 9/3-minute Stryd CP test and the race's target CP (37). Supporting this, Smyth and Muniz-Pumares (41) demonstrated that runners initiating a marathon at approximately 87.6% of their CS generally achieve better outcomes, whereas those starting above this threshold often experience pronounced slowdowns as the race progresses. Similarly, marathon speeds average around 84.8% of CS, with faster runners competing closer to their CS (∼93%) compared to slower runners (∼78.9%). These findings highlight CS's versatility not only as a robust predictor of performance but also as a valuable tool for refining pacing strategies across different performance levels. By tailoring race strategies based on CS, runners can better balance energy expenditure and mitigate fatigue, ultimately enhancing race outcomes.

The application of CS is not limited to race-day strategies but extends to training program design. Field-based CS tests, such as TTs and 3MT, provide reliable metrics that coaches can use to design individualized training programs. Intervals prescribed just below CS help optimize aerobic endurance, while those above CS engage anaerobic capacity. Clark et al. (58) demonstrated that high-intensity interval training (HIIT) prescribed at intensities between 110% and 130% of CS has been shown to yield significant improvements in aerobic performance. Moreover, enhancing CS can translate directly into improved race results. For example, increasing CS from 4.90 m/s to 4.99 m/s has been shown to result in a 36 s reduction in a 10,000 m race time, underscoring the substantial impact of targeted CS training program (35).

By integrating CS into training and race planning, runners and coaches can better manage pacing, fatigue, and energy reserves, leading to optimized performance. These applications highlight CS's critical role in bridging theoretical models and practical strategies, making it an indispensable tool for endurance athletes aiming to achieve peak performance.

### 4.4 Practical summary of field tests

Field-based testing methods offer significant advantages for a wide range of athletes, including their simplicity, accessibility, and suitability for various performance levels. For recreational runners, tests can often be performed using minimal equipment, such as a stopwatch and a marked distance, and completed within a single day with a 30-min recovery period. However, several practical factors must be considered to ensure test reliability and validity.

To aid practitioners in implementing these methods effectively, Table 4 provides a summary of key recommendations and limitations for different protocols, including TTs, TTE, 3MT, and approaches based on raw or habitual training data. This table highlights critical parameters such as test duration, recovery times, environmental considerations, and equipment requirements, offering a practical guide for optimizing test design and execution.

**Table 4:** Comparison of field-based testing testing methods for critical speed estimation.

Method	Factor	Key recommendation	Limitation
TTs	Length, duration	Use fixed durations or distances (e.g., 3, 7, and 12 min or 1,200, 2,400, 3,600 m), Trials should be 3–12 min to balance aerobic and anaerobic energy systems.	Shorter trials (<3 min) rely heavily on anaerobic systems; longer trials (>15 min) risk reduced intensity.
	Number of trials	Minimum of two trials (e.g., 1,200 m and 3,600 m) for practical settings; three trials for increased precision.	Two trials may not fully account for D′ variability or standard error.
	Recovery time	Recovery: 30–60 min between trials; random or longest-to-shortest order.	Short recovery times may affect fatigue and performance reliability.
	Environmental Factors	Perform tests in consistent conditions (e.g., track or flat terrain).	Requires high motivation and pacing consistency; environmental conditions can introduce variability.
	Equipment	Stopwatch, GPS devices or power meters for accurate data collection (e.g., Stryd power meter).	Device placement (e.g., left vs. right foot) can introduce variability in data. (Stryd device). Availability and cost of devices like GPS or Stryd may limit accessibility in some settings.
	Verification of VO_2max_	Optional but enhances reliability; consider portable metabolic analyzers for field tests.	Adds cost and complexity; limited availability in some settings.
TTE	Duration	Trials between 2 and 15 min with consistent pacing	Challenging to maintain consistent pacing without physiological feedback (e.g., VO_2max_ verification).
	Number of trials	3–5 trials to improve reliability.	Time-consuming; impractical in some field settings.
	Environmental factors	Perform tests in controlled and consistent conditions.	Weather, wind, and surface variability may influence reliability.
	Equipment	auditory tone for speed synchronization	Dependence on auditory cues may not fully eliminate pacing variability or ensure maximal effort.
	Verification of VO_2max_	Optional but enhances reliability; consider portable metabolic analyzers for VO_2max_ validation.	Adds cost and complexity; not always feasible in field settings.
3MT	Protocol design	Perform a single all-out effort to deplete anaerobic reserve and estimate CS.	May underestimate D′ by ∼16%; maximal effort varies among participants.
	Environmental factors	Conduct tests in consistent conditions (e.g., track or flat terrain).	Weather, surface, or temperature may influence results.
	Equipment	Use GPS devices, accelerometers, or power meters (e.g., Stryd) for real-time monitoring and data collection.	Device placement (e.g., left vs. right foot) can introduce variability in data. (Stryd device). Availability and cost of devices like GPS or Stryd may limit accessibility in some settings.
	Participant suitability	Best suited for experienced runners accustomed to maximal intensity efforts.	Less practical for novices or those unaccustomed to all-out tests.
Raw/habitual data approach	Data source	Use recent race data, or habitual training data for CS estimation.	Relies on consistent effort and accurate data tracking; maximal effort in races may vary by motivation.
	Duration	Combine data from multiple races/training (e.g., 400, 800, 5,000 m or 3, 7, 12 min)	Questions remain about the optimal duration of HAB data collection.
	Environmental factors	Ensure consistent training/race conditions where possible.	Variability in conditions (e.g., wind, terrain) may affect results.
	Equipment	Use GPS devices or power meters to record and analyze data trends.	Data accuracy depends on device calibration and consistent use.
			Availability and cost of devices like GPS or Stryd may limit accessibility in some settings

CS, critical speed; D′, the finite distance above CS; TTs, time trials; TTE, time to exhaustion; 3MT, 3 min all-out test; GPS, global positioning system; VO_2max_, maximal oxygen uptake; HAB, habitual training data.

## 5 Study limitations

This review has some limitations. First, this study is not a meta-analysis, which limits the depth of statistical analysis and generalization of the results. Additionally, comparing different protocols presents challenges, as they are not fully standardized and can vary in key aspects. Another limitation is that most of the included studies focused on participants with running experience, making it difficult to determine how these findings would apply to the general population or recreational athletes. Furthermore, the studies reviewed were predominantly male-based, resulting in a gender imbalance that may affect the generalizability of the findings. Lastly, the findings of this review should be interpreted with caution due to the potential risk of bias in the included studies.

## 6 Conclusion

This systematic review highlights the effectiveness of field-based assessments, such as TTs and the 3MT, for determining CS in runners. These protocols have shown practical utility in real-world conditions, particularly for predicting outdoor race performance. Key factors influencing the accuracy of CS include the number and duration of trials, recovery time, and trial order. With their ecological validity, field-based methods offer a more practical and relevant approach for performance assessment compared to laboratory-based tests, particularly in experienced athletes. Emerging approaches, such as the use of raw race data or habitual training data, hold significant promise for simplifying field-based testing and seamlessly integrating it into everyday training practices. These methods, while still in the early stages of exploration, could greatly enhance accessibility and applicability across diverse athletic populations and environments. A major gap in the current research lies in the limited inclusion of female participants, which raises questions about the generalizability of findings. Future studies should prioritize addressing this disparity to ensure that protocols are equally effective and applicable for athletes of all genders. Additionally, continued efforts are needed to refine data-driven methods and explore their potential to revolutionize field-based assessments, enabling more personalized and adaptive performance evaluations.

## Data Availability

The original contributions presented in the study are included in the article/Supplementary Material, further inquiries can be directed to the corresponding author.
