# Supplementary material for: Field-based tests for determining critical speed among runners and its practical application: a systematic review
Source: Front Sports Act Living. 2025 Mar 11;7:1520914. doi: 10.3389/fspor.2025.1520914 (PMC11933073; doi:10.3389/fspor.2025.1520914)
Supplement: Supplementary file 1 [file Table1.docx]

Supplementary Material

Supplementary material - Field-Based Tests for Determining Critical Speed among Runners and its Practical Application: A Systematic Review

Table S1: Customize Downs and Black scale

| Item | Criteria | Possible Answers |
| --- | --- | --- |
| Reporting | | |
| 1 | *Is the hypothesis/aim/objective of the study clearly described?* This item is only rated as a Yes, if both aim/purpose and hypothesis are described. In case the study design does not allow any hypothesis or the direction of the study is so novel that no prior hypothesis can be formed, and this is made clear in the introduction, the item should be rated as a Yes. | Yes = 1  No = 0 |
| 2 | *Are the main outcomes to be measured clearly described in the Introduction or Methods section?* If the main outcomes are first mentioned in the Results section, the question should be answered no. | Yes = 1  No = 0 |
| 3 | *Are the characteristics of the participants included in the study clearly described?* If there is detailed description of the athletes (level, type of sport, age, gender, experience/training volume), the item is marked as a Yes. | Yes = 1  No = 0 |
| 4 | *Are the methods of interest clearly described?* If the methods are all clear described, the item is marked as a Yes. | Yes = 1  No = 0 |
| 6 | *Are the main findings of the study clearly described?* In this review, validity and reliability of CP/CS or comparison/estimation of different methods are the major findings. | Yes = 1  No = 0 |
| 7 | *Does the study provide estimates of the random variability in the data for the main outcomes?* In non-normally distributed data the interquartile range of results should be reported. In normally distributed data the standard error, standard deviation or confidence intervals should be reported. If the distribution of the data is not described, it must be assumed that the estimates used were appropriate and the question should be answered yes. | Yes = 1  No = 0 |
| 10 | *Have actual probability values been reported (e.g. 0.035 rather than <0.05) for the main outcomes except where the probability value is less than 0.001?* Does the study present p-value e.g. 0.035 rather than <0.05 for  the  main  outcomes  except where the probability value is less than 0.001? Rate Yes if the study includes this criterion. | Yes = 1  No = 0 |
| External validity | | |
| 11 | *Were the subjects asked to participate in the study representative of the entire population from which they were recruited?*  The study must identify the source population for participants and describe how the participants were selected. Participants would be representative if they comprised the entire source population, an unselected sample of consecutive participants, or a random sample. Random sampling is only feasible where a list of all members of the relevant population exists. Where a study does not report the proportion of the source population from which the patients are derived, the question should be answered as unable to determine. The source population in our review is defined as female athletes in the respective sports. | Yes = 1  No = 0  Unable to determine = 0 |
| 12 | *Were those subjects who were prepared to participate representative of the entire population from which they were recruited?*  The proportion of those asked who agreed should be stated. Validation that the sample was representative would include demonstrating that the distribution of the main confounding factors was the same in the study sample and the source population. | Yes = 1  No = 0  Unable to determine = 0 |
| Internal validity – bias | | |
| 18 | *Were the statistical tests used to assess the main outcomes appropriate?* The statistical techniques used must be appropriate to the data. For example nonparametric methods should be used for small sample sizes. Where little statistical analysis has been undertaken but where there is no evidence of bias, the question should be answered yes. If the distribution of the data (normal or not) is not described it must be assumed that the estimates used were appropriate and the question should be answered yes*.* | Yes = 1  No = 0  Unable to determine = 0 |
| 20 | *Were the main outcome measures used accurate (valid and reliable)?* For studies where the outcome measures are clearly described, the question should be answered yes. For studies which refer to other work or that demonstrates the outcome measures are accurate, the question should be answered as yes. | Yes = 1  No = 0  Unable to determine = 0 |
| 21 | *Were the participants in different intervention groups (trials and cohort studies) or were the cases and controls (case-control studies) recruited from the same population?*  If the participants in all groups were recruited from the same target population, the answer is Yes. | Yes = 1  No = 0  Unable to determine = 0 |
| 22 | *Were study participants in different intervention groups (trials and cohort studies) or were the cases and controls (case-control studies) recruited over the same period of time?*  If the participants were recruited during the same time period, the answer is Yes. | Yes = 1  No = 0  Unable to determine = 0 |
| 25 | *Was there an adequate adjustment for confounding in the analyses from which the main findings were drawn?*  This item should be rated as a Yes if the study explicitly accounts for key factors influencing CS results. Verification should include at least one of the following: Consistent testing protocols (e.g., identical trial distances or durations across participants); Control of environmental conditions (e.g., track vs. outdoor terrain); Standardization of participant characteristics (e.g., training level, fatigue state). | Yes = 1  No = 0  Unable to determine = 0 |
| 26 | *Were losses of patients to follow‐up taken into account?*  If the numbers of athletes` losses are not reported, the question should be answered as unable to determine. If the proportion lost was too small to affect the main findings, the question should be answered yes. | Yes = 1  No = 0  Unable to determine = 0 |
| Power | | |
| 27 | 27. *Did the study have sufficient power to detect a clinically important effect where the probability value for a difference is due to chance is less than 5%?*    Sample size calculation performed = yes |  |
