# Supplementary material for: Field-based tests for determining critical speed among runners and its practical application: a systematic review
Source: Front Sports Act Living. 2025 Mar 11;7:1520914. doi: 10.3389/fspor.2025.1520914 (PMC11933073; doi:10.3389/fspor.2025.1520914)
Supplement: Supplementary file 2 [file Table2.docx]

Supplementary Material

Supplementary material - Field-Based Tests for Determining Critical Speed among Runners and its Practical Application: A Systematic Review

Table S2: Assessment of Study Quality

|  | **Authors** | **1** | **2** | **3** | **4** | **6** | **7** | **10** | **11** | **12** | **18** | **20** | **21** | **22** | **25** | **26** | **27** | **Together** |
| --- | --- | --- | --- | --- | --- | --- | --- | --- | --- | --- | --- | --- | --- | --- | --- | --- | --- | --- |
| 1 | Aguiar | 1 | 1 | 1 | 1 | 0 | 1 | 1 | 0 | 0 | 1 | 1 | 1 | 1 | 1 | 1 | 0 | 11/15 |
| 2 | Broxterman | 1 | 1 | 1 | 1 | 1 | 1 | 1 | 0 | 0 | 1 | 1 | 1 | 1 | 1 | 1 | 0 | 12/15 |
| 3 | Correa | 1 | 1 | 1 | 1 | 0 | 1 | 0 | 0 | 0 | 1 | 1 | 1 | 1 | 0 | 0 | 0 | 8/15 |
| 4 | Figueiredo | 1 | 1 | 1 | 1 | 1 | 1 | 0 | 0 | 0 | 1 | 1 | 1 | 1 | 1 | 0 | 0 | 10/15 |
| 5 | Galbraith 2014 | 1 | 1 | 1 | 1 | 1 | 1 | 0 | 0 | 0 | 1 | 1 | 1 | 1 | 0 | 0 | 0 | 9/15 |
| 6 | Galbraith 2015 | 1 | 1 | 0 | 1 | 1 | 1 | 0 | 0 | 0 | 1 | 1 | 1 | 1 | 1 | 0 | 0 | 9/15 |
| 7 | Galbraith 2014 | 1 | 1 | 1 | 1 | 1 | 1 | 1 | 0 | 0 | 1 | 1 | 1 | 1 | 1 | 0 | 0 | 11/15 |
| 8 | Hunter 2023 | 1 | 1 | 1 | 1 | 1 | 1 | 1 | 0 | 0 | 1 | 1 | 1 | 1 | 1 | 0 | 1 | 12/15 |
| 9 | Kordi | 1 | 1 | 1 | 1 | 0 | 1 | 0 | 1 | 0 | 1 | 1 | 1 | 1 | 1 | 0 | 0 | 10/15 |
| 10 | Olaya-Cuartero | 1 | 1 | 1 | 1 | 1 | 1 | 1 | 0 | 0 | 1 | 1 | 1 | 1 | 1 | 0 | 0 | 11/15 |
| 11 | Pettitt | 1 | 1 | 0 | 1 | 0 | 1 | 0 | 0 | 0 | 1 | 1 | 1 | 1 | 1 | 0 | 0 | 8/15 |
| 12 | Ribeiro | 1 | 1 | 0 | 1 | 1 | 1 | 0 | 0 | 0 | 1 | 1 | 0 | 1 | 1 | 0 | 0 | 8/15 |
| 13 | Ruiz 2023a | 1 | 1 | 1 | 1 | 1 | 1 | 1 | 1 | 0 | 1 | 1 | 1 | 1 | 1 | 0 | 0 | 12/15 |
| 14 | Ruiz 2023b | 1 | 1 | 1 | 1 | 1 | 1 | 1 | 1 | 0 | 1 | 1 | 1 | 1 | 1 | 0 | 0 | 12/15 |
| 15 | Smyth | 1 | 1 | 1 | 1 | 1 | 1 | 1 | 1 | 0 | 1 | 1 | 1 | 1 | 0 | 0 | 0 | 11/15 |
| 16 | Triska 2018 | 1 | 1 | 1 | 1 | 0 | 1 | 1 | 0 | 0 | 1 | 1 | 1 | 1 | 1 | 0 | 0 | 10/15 |
| 17 | Triska 2017 | 1 | 1 | 1 | 1 | 0 | 1 | 0 | 0 | 0 | 1 | 1 | 1 | 1 | 1 | 0 | 0 | 9/15 |
| 18 | Van Rassel | 1 | 1 | 1 | 1 | 1 | 1 | 0 | 0 | 0 | 1 | 1 | 1 | 1 | 1 | 0 | 0 | 10/15 |
| 19 | Vassallo | 1 | 1 | 1 | 1 | 1 | 1 | 1 | 1 | 0 | 1 | 1 | 1 | 1 | 1 | 0 | 0 | 12/15 |
